# Stress echocardiographic assessment of mitral valve function repaired using rough-zone trimming

**DOI:** 10.1186/s13019-015-0232-y

**Published:** 2015-02-28

**Authors:** Yohsuke Yanase, Nobuyuki Takagi, Hiroyuki Yamada, Toshitaka Watanabe, Mayuko Uehara, Kazutoshi Tachibana, Yasuko Miyaki, Toshiro Ito, Tetsuya Higami

**Affiliations:** Departmetnt of Cardiovascular Surgery, Sapporo Medical University of Medicine, South 1, West 16, Chuo-ku, Sapporo, Hokkaido 060-8543 Japan

**Keywords:** Mitral regurgitation, Mitral valve repair, Echocardiography, Heart valve, Surgery techniques

## Abstract

**Background:**

We invented novel mitral valve repair technique; rough-zone trimming procedure (RZT) for anterior mitral valve prolapse. Prolapse site was resected in obtuse triangle shape and sutured edges to creates deep coaptation and improves regurgitation. Though it is simple and reproducible technique, functional mitral stenosis is a risk. Valve function and hemodynamics were investigated using dobutamine stress echocardiography (DSE) in patients after mitral valve repair using RZT.

**Methods:**

Patients underwent RZT for the anterior mitral valve (AML, n = 10), quadrangular resection (QR) of the posterior mitral valve (PML; n = 4), RZT + QR of bileaflet valves (bileaflet; n = 4) and healthy individuals (control; n = 10) and were assessed by DSE (doses up to 20 μg/Kg/min). Echocardiographic data including mitral valve area (MVA), mitral valve mean pressure gradient (MVmeanPG), and systolic pulmonary artery pressure (sPAP) were measured at rest and at peak stress.

**Results:**

Rest/stress MVA (cm^2^), MVmeanPG (mmHg) and sPAP (mmHg) were 2.8 ± 0.4 and 3.4 ± 0.3, 3.3 ± 1.1 and 7.4 ± 4.1, and 25.7 ± 4.7and 49.1 ± 4.1, respectively, in the AML group. Dobutamine stress increased all parameters but not to pathological levels. The results were similar to those of the other groups after mitral valve repair, whereas MVA was larger and MVmeanPG was lower in the control than in the AML group.

**Conclusions:**

Valve repair using RZT does not pathologically obstruct the mitral valve, either at baseline or during dobutamine stress, and does not affect valve hemodynamics and reserve.

## Background

### Introduction

**Figure 1:**
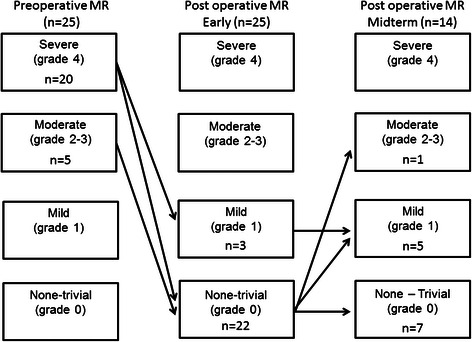
**Preoperative and postoperative echocardiographic grade of mitral regurgitation (MR).** Preoperative MR grades were severe and moderate 20 and 5 patients, respectively. Mitral valves were repaired using rough-zone trimming (RZT). All grades improved to below mild MR grades within early postoperative period of 30 days. By mid-term (>12 months), 13 of 14 patients had better than mild MR. Only one patient worsened to moderate MR because of progressive sclerotic valve changes.

Mitral valve repair rather than replacement has become the first choice of surgical treatment for mitral valve regurgitation because unnecessary of anticoagulant therapy and restoration of left ventricular function. Several techniques to repair the mitral valve have been presented and we invented a novel mitral valve repair technique (rough-zone trimming: RZT) to treat anterior mitral valve prolapse. The prolapse site is resected in RZT within a small obtuse triangle shape limited to the rough zone and re-sutured edges. This creates deep coaptation and prevents regurgitation. Among repaired mitral valves in 96 patients between February 2007 and January 2013, 49 of them were repaired using RZT (diseased anterior mitral leaflet, n = 25; diseased bileaflet, n = 24). The early and midterm (>12 months) outcomes of RZT for the repair of the anterior mitral leaflet were satisfactory (Figure 1).

### Purpose

Although repair of an anterior mitral leaflet prolapse remains challenging, RZT has generated reproducible and reliable results. But functional mitral stenosis remains a potential risk. Here, we applied dobutamine stress echocardiography (DSE) to assess the hemodynamics of mitral valves that were repaired using RZT.

## Methods

### Patients

All patients underwent mitral valve repair of anterior leaflets between February 2007 and January 2013. Patients who underwent mitral valve repair for posterior leaflets, bileaflets and healthy controls were also included. The elimination criteria comprised serious disorders such as angina pectoris, severe hypertension, arrhythmia, cerebral bleeding, aortic aneurysm, aortic dissection, < 6 months after mitral valve repair and withholding written consent to participate. Our institutional review board and medical ethics committee approved the present study (approval number: 25-2-40 and 25–1147).

### Surgical procedure

Cardiopulmonary bypass was established via a full median sternotomy, the ascending aorta, and the superior and inferior vena cava were directly cannulated, the ascending aorta was cross-clamped, and then antegrade and retrograde cardioplegic solution was injected. The mitral valve was exposed via a conventional left atriotomy.

**Figure 2:**
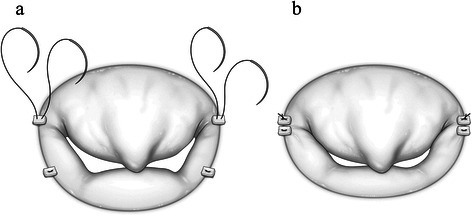
**Schema of enhanced stay sutures (ESS).** Mitral valve leaflet and medial site of anterior leaflet is elongated and prolapsed. **(a)** Horizontal mattress sutures (3–0 polyester suture with pledgets) for anterior and posterior commissures. Stitches pulled bigger bites out of posterior annular side. **(b)** After ESS. Anterior and posterior commissures are compressed and coaptation is deeper. ESS allowed not only deeper coaptation, but also pulled posterior leaflet upwards to the same plane as anterior leaflet by taking bigger bites out of the posterior annular side. This also rendered prolapse sites highly visible.

The mitral valve configuration and the prolapse site were checked using saline injection. Anterior and posterior mitral commissures were compressed using horizontal mattress sutures (3–0 polyester with pleget). We ensured that the stitch was bigger on the posterior annular side, which led to not only deeper leaflet coaptation, but also the ability to draw the posterior leaflet upwards to the same plane as the anterior leaflet. Thus, the prolapse site became obvious. The technique is similar to Kay sutures [1], but the concept slightly differs, and we named it the enhanced stay suture (ESS; Figure 2). After ESS, details of prolapse sites were reconfirmed by saline injection and the mitral valve was repaired.

### Mitral valve repair for anterior leaflet

**Figure 3:**
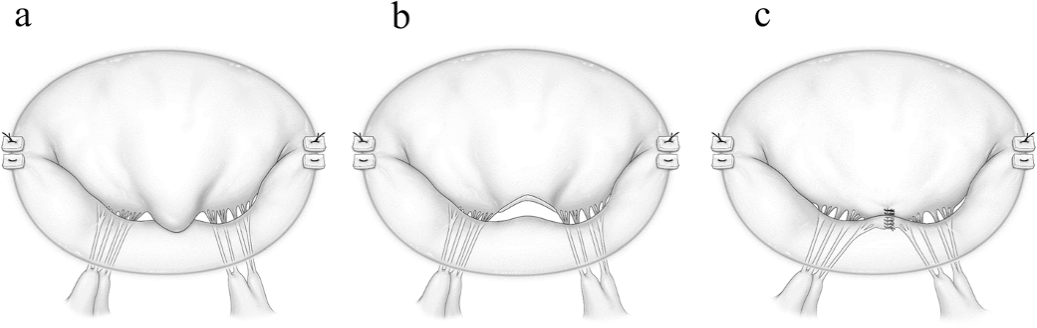
**Schema of rough-zone trimming (RZT). (a)** Medial scallop (A2) of anterior mitral valve leaflet (AML) is elongated and prolapsed after previous ESS. **(b)** Prolapsed A2 site resected in shape of obtuse triangle limited to rough-zone. **(c)** Resected edges repaired with 6–0 monofilament sutures. Repaired site of valve leaflet was taken down into left ventricular side and deeper coaptation improved mitral valve regurgitation.

We used the RZT to repair prolapse sites that were located in the anterior leaflet. Prolapsed sites were resected in the shape of an obtuse triangle and confined to the rough zone. The resected height of the triangle is confined to the rough-zone (not cut into the clear zone). The resected base length of the triangle is only the degenerated and thickened area. Resected edges were then brought together with interrupted 6–0 monofilament sutures. The repaired site of the valve leaflet was brought down into the left ventricular side to acquire deeper coaptation, which improved mitral valve regurgitation. (RZT; Figure 3).

### Mitral valve repair for posterior leaflet

We repaired prolapse sites in posterior leaflets using quadrangular resection (QR) [2]. The posterior annulus was stitched using 3–0 polyester pledgeted compression sutures and resected edges were closed with interrupted 6–0 monofilament sutures.

### Mitral valve repair for anterior combination with posterior leaflet

We repaired prolapse sites in the anterior and posterior leaflets (bileaflet) using both RZT and QR techniques.

After repairing mitral valve leaflets, annuloplasty proceeded using a suitable flexible band to prevent future annular dilation. Since the annular size was adjusted by ESS, we did not apply annular plication with the flexible band. After completing a series of valve repairs procedures, we performed retrograde cardioprotective beating test (RC-beating test) to check for residual regurgitation [3]. Under aortic cross-clamping and before closing the left atrium, cardiac perfusion was retrogradely started via the coronary sinus and cardiac beating was started to directly visualize and evaluate residual regurgitation. Repair of the mitral valve repair was completed after the absence of leakage was confirmed.

### Echocardiography

One investigator evaluated all patients using a Phillips iE33 ultrasound device (Royal Philips Electronics, Amsterdam, Netherlands). Left ventricular diameters (LVEDD, LVESD), volumes (LVEDV, LVESV), ejection fraction (EF) derived from the Teichholz and Simpson rule. Cardiac output was calculated as stroke volume (LVEDV - LVESV) × heart rate (HR). The mitral valve area (MVA) was calculated using the pressure half-time (PHT) method. Mean and peak transmitral pressure gradients (MVmeanPG, MVmaxPG) were determined using continuous wave Doppler echography. Systolic pulmonary pressure (sPAP) was derived from the maximal velocity of the tricuspid Doppler tracing at a fixed right atrial pressure of 10 mmHg. The degree of mitral regurgitation (MR) was estimated from the maximum regurgitant jet area (MRA) in color flow Doppler echocardiograms.

The protocol for dobutamine stress echocardiography is described below. All patients were placed in the left decubitus position for echocardiographic assessment at rest. Thereafter, the DSE study was started by intravenously infusing dobutamine (5 μg/Kg/min) using a syringe driver. Blood concentration were allowed to stabilize for five minutes and then the first stage of the echocardiographic study started by increasing the dobutamine dose in 5 μg/Kg/min increments every three minutes to a maximum of 10, 15 and 20 μg/Kg/min. Echocardiographic data were collected at each stage. Blood pressure, oxygen saturation and electrocardiographic findings were continuously monitored throughout the study, which was stopped when either of the following end-points was achieved: target heart rate of > 85% of maximum predicted (220 - age) or the dobutamine dose was maximal (20 μg/Kg/min). The study was discontinued if any of the following events occurred: intolerable symptoms (chest pain or progressive dyspnea), limiting asymptomatic side effects such as systolic or diastolic blood pressure of > 220 and/or > 120 mmHg, hypotension defined as a relative or absolute > 30 mmHg decrease blood pressure, supraventricular tachycardia or atrial fibrillation, ventricular tachycardia or frequent, polymorphous premature ventricular beats. Acquired data were verified according to the American College of Cardiology/American Heart guidelines for the management of patients with valvular heart disease (2008) [4] in which MVA < 1.0 cm^2^, or MVmeanPG under stress of > 15 mmHg, or sPAP under stress of > 60 mmHg were defined as severe mitral stenosis requiring surgery.

The severity of MR was classified as none or trivial (MRA ≤ 2.0 cm^2^; grade 0), mild (MRA >2.0 ≤ 4.0 cm^2^; grade 1), moderate (MRA > 4 ≤ 6 cm^2^; grade 2), moderately severe (MRA > 6 ≤ 8 cm^2^; grade 3) and severe (MRA > 8 cm^2^; grade 4) [5].

### Statistical analysis

All values are expressed as means ± standard deviation. Rest and stress data were compared using a paired *t*-test. Between-group comparisons were analyzed using Welch’s *t*-test. P < 0.05 was considered statistically significant.

## Results

Nineteen patients with repaired mitral valves and 11 healthy control individuals participated in the DSE study. Paroxysmal supraventricular tachycardia developed in one patient after mitral valve repair for the anterior leaflet and accelerated ideo-ventricular rhythm developed in one healthy control. Both opted out of the study and their data were excluded. Therefore, the DSE study was completed in 10 patients in the AML group, four each in the PML and bileaflet groups and in 10 healthy controls (Table 1) and implemented at an average of 20.7 (7.2-40.9), 32.1(9.2-54.3) and 22.4 (7.3 - 35.0) months in these groups, respectively.

**Table 1:** **Patients’ characteristics**

	AML (n = 10)	PML (n = 4)	Bileaflet (n = 4)	Control (n = 10)
Sex (M:F) (n)	8:2	4:0	3:1	10:0
Age (y)	56.5 ± 19.3	59.0 ± 6.1	53.3 ± 6.1	25.0 ± 3.7
Infective endocarditis (n)	1	0	1	-
Annuloplasty ring size (mm)				
26 (n)	2	1	1	-
27 (n)	0	1	0	-
28 (n)	3	1	2	-
30 (n)	1	1	1	-
No annuloplasty	4	0	0	-
Residual mitral regurgitation				
None-Trivial (grade 0) (n)	9	4	3	-
Mild (grade 1) (n)	1	0	1	-

AML, anterior mitral leaflet; F, female; M, male; PML, posterior mitral leaflet.

**Figure 4:**
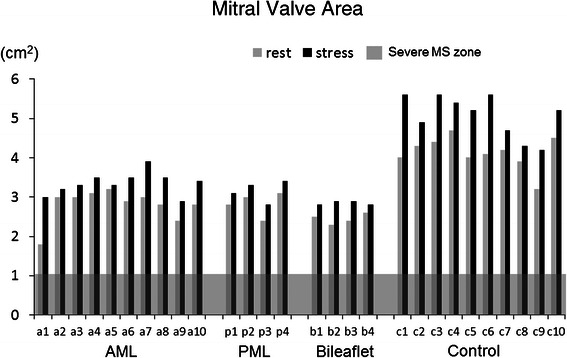
**Echocardiographic data of mitral valve area (MVA).** Echocardiographic data of mitral valve area (MVA) at rest and peak stress in AML, PML, bileaflet and control groups.

**Figure 5:**
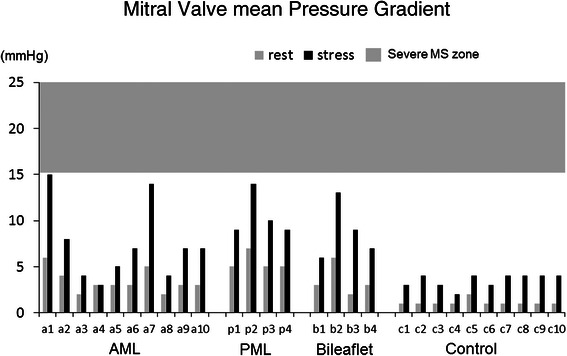
**Echocardiographic data of mitral valve mean pressure gradient (MVmeanPG).** Echocardiographic data of mitral valve mean pressure gradient (MVmeanPG) at rest and peak stress in AML, PML, bileaflet and control groups.

**Figure 6:**
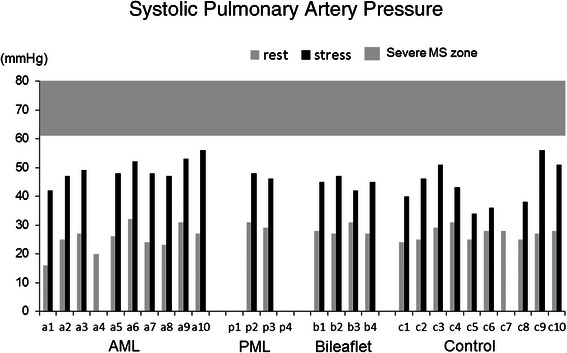
**Echocardiographic data of systolic pulmonary artery pressure (sPAP).** Echocardiographic data of systolic pulmonary artery pressure (sPAP) at rest and peak stress in AML, PML, bileaflet and control groups.

The degree of MR was below mild in all patients at rest and did not worsen in any of them with stress. Systolic anterior movement (SAM) also did not appeared at rest and with stress in all patients. Hemodynamic data such as heart rate, systolic blood pressure and cardiac output were significantly increased in all groups. The rest/stress values for MVA (cm^2^) in the AML, PML, bileaflet and control groups were 2.8 ± 0.4/3.4 ± 0.3, (p = 0.0005), 2.8 ± 0.3/3.2 ± 0.3, (p = 0.001), 2.5 ± 0.1/2.9 ± 0.1, (p = 0.022) and 4.1 ± 0.4/5.1 ± 0.5, (p = 0.0001), respectively (Figure 4). The MVmeanPG (mmHg) in the AML, PML, bileaflet and control groups were 3.3 ± 1.1/7.4 ± 4.1, (p = 0.0026); 5.5 ± 1.0/10.5 ± 2.4, (p = 0.0058); 3.5 ± 1.7/8.8 ± 3.1, (p = 0.0146) and 1.1 ± 0.3/3.6 ± 0.7, (p < 0.0001), respectively (Figure 5). The sPAP (mmHg) in the AML, PML, bileaflet and control groups were 25.7 ± 4.7/49.1 ± 4.1, (p < 0.0001); 30.0 ± 1.4/47.0 ± 1.4; 28.3 ± 1.9/44.8 ± 2.1, (p = 0.0034) and 26.9 ± 2.3/43.9 ± 7.6, (p = 0.0001); respectively (Figure 6). The MVA, MVmeanPG and sPAP were significantly increased at peak stress in all groups (Table 2), but severe functional mitral stenosis requiring re-operation was not identified in any of the groups.

**Table 2:** **Hemodynamic data at rest and peak stress during dobutamine stress echocardiography**

Variable	AML (n = 10)			PML (n = 4)			Bileaflet (n = 4)			Control (n = 10)		
	Rest	Stress	p	Rest	Stress	p	Rest	Stress	p	Rest	Stress	p
Mitral valve area (cm^2^)	2.8 ± 0.4	3.4 ± 0.3	0.0005	2.8 ± 0.3	3.2 ± 0.3	0.0010	2.5 ± 0.1	2.9 ± 0.1	0.0220	4.1 ± 0.4	5.1 ± 0.5	0.0001
MVmaxPG (mmHg)	9.2 ± 3.0	16.3 ± 6.0	0.0079	12.0 ± 2.5	22.3 ± 5.0	0.0077	9.3 ± 4.2	17.5 ± 4.7	0.0071	3.8 ± 0.9	9.4 ± 3.7	0.0003
MVmeanPG (mmHg)	3.3 ± 1.1	7.4 ± 4.1	0.0026	5.5 ± 1.0	10.5 ± 2.4	0.0058	3.5 ± 1.7	8.8 ± 3.1	0.0146	1.1 ± 0.3	3.6 ± 0.7	0.0222
sPAP (mmHg)	25.7 ± 4.7	49.1 ± 4.1	0.0000	30 ± 1.4	47.0 ± 1.4	^a^	28.3 ± 1.9	44.8 ± 2.1	0.0034	26.9 ± 2.3	43.9 ± 7.6	0.0001
MR grade (mean)	0.1 ± 0.3	0.1 ± 0.3	-	0	0	-.	0.3 ± 0.5	0	0.3910	0	0	-
HR (beats/min)	73 ± 16	107 ± 24	0.0002	76 ± 2	127 ± 25	0.0225	68 ± 3	111 ± 19	0.0138	64 ± 11	115 ± 16	0.0000
sBP (mmHg)	115 ± 13	150 ± 20	0.0008	127 ± 14.0	143.3 ± 26.5	0.1495	115 ± 14	145 ± 33	0.1991	104 ± 8	153 ± 18	0.0000
dBP (mmHg)	69 ± 13	71 ± 13	0.5426	76.3 ± 9.2	71 ± 6.9	0.1261	69 ± 16	75 ± 17	0.6440	53 ± 9	65 ± 9	0.0069
CO (L/min)	4.4 ± 1.0	7.9 ± 1.4	0.0000	5.0 ± 0.4	9.6 ± 2.5	0.0234	4.2 ± 0.3	7.5 ± 1.0	0.0023	3.9 ± 0.7	9.2 ± 1.4	0.0000
EF (%)	64.7 ± 2.9	81.9 ± 2.5	0.0000	61.9 ± 6.7	81.9 ± 4.5	0.0006	62.8 ± 1.5	83.9 ± 4.7	0.0045	62.6 ± 3.6	82.0 ± 2.7	0.0000
LVEDV (ml)	92.5. ± 10.3	91.3 ± 8.0	0.5931	107.9 ± 19.6	89.4 ± 13.5	0.0262	98.3 ± 10.7	82.7 ± 16.2	0.0323	99.6 ± 13.4	99.0 ± 14.0	0.8726
LVESV (ml)	31.7 ± 4.7	16.6 ± 3.4	0.0000	42.3 ± 15.0	15.0 ± 4.6	0.0195	36.5 ± 3.7	13.9 ± 5.7	0.0015	38.0 ± 6.7	18.1 ± 5.4	0.0000

AML, anterior mitral leaflet; CO, cardiac output; dBP, diastolic blood pressure; EF, ejection fraction; LVEDV, left ventricular end diastolic volume; LVESV, left ventricular end systolic volume; MR, mitral valve regurgitation; MVmaxPG, mitral valve maximum pressure gradient; MVmeanPG, mitral valve mean pressure gradient; p, p value; PML, posterior mitral leaflet; sBP, systolic blood pressure; sPAP, systolic pulmonary artery pressure. ^a^PML sPAP data were not statistically analyzed due to small sample size.

We compared rest/stress MVA, MVmeanPG and sPAP between the AML and the other groups. Though the stress sPAP was lower for the Bileaflet group than for the AML group, other data did not show hemodynamic disadvantage of the AML group compare with other mitral valve repair groups (Table 3). The rest/stress MVA was significantly larger for the control than for the AML group. The rest/stress MVmeanPG was significantly lower for the control than for the AML group. Otherwise sPAP did not significantly differ between the AML and control groups.

**Table 3:** **Echocardiographic comparison between AML and other groups that underwent mitral valve repair**

	Variable	AML	PML	Bileaflet	Control
**Rest**	**MVA (cm** ^**2**^ **)**	2.8 ± 0.4	2.8 ± 0.3	2.5 ± 0.1^a^	4.1 ± 0.4^b^
	**MVmeanPG (mmHg)**	3.3 ± 1.1	5.5 ± 1.0^a^	3.5 ± 1.7	1.1 ± 0.3^b^
	**sPAP (mmHg)**	25.7 ± 4.7	30 ± 1.4^a^	28.3 ± 1.9	26.7 ± 2.3
	**Variable**	**AML**	**PML**	**Bileaflet**	**Control**
**Stress**	**MVA (cm** ^**2**^ **)**	3.4 ± 0.3	3.2 ± 0.3	2.9 ± 0.1^b^	5.1 ± 0.5^b^
	**MVmeanPG (mmHg)**	7.4 ± 4.1	10.5 ± 2.4	8.8 ± 3.1	3.6 ± 0.7^a^
	**sPAP (mmHg)**	49.1 ± 4.1	47.0 ± 1.4	44.8 ± 2.1^a^	43.9 ± 7.6

AML, anterior mitral leaflet; MVA, mitral valve area; MVmeanPG, mitral valve mean pressure gradient; PML, posterior mitral leaflet; sPAP, systolic pulmonary artery pressure. ^a^p < 0.05 and ^b^p < 0.01 vs. AML.

## Discussion

### Summary of current mitral valve repair

Mitral valve repair has become the preferred surgical treatment for MR and although previously challenging for treating the diseased anterior leaflet [6], newer methods such as artificial chordal implantation [7] and the edge-to-edge method [8] have improved outcomes [9,10]. However, some technical difficulties such as adjusting the length of artificial chordae during implantation and mitral stenosis are concerns in the edge-to-edge method. We overcame these problems using the simple and reproducible RZT with ESS.

### Novel mitral valve repair technique “Rough-zone trimming & Enhanced stay suture”

The conventional triangular resection procedure cuts into the clear zone of the anterior leaflet and resects a large leaflet area. Thus, there is a risk of dehiscence of suture line and severe mitral stenosis. In RZT, since the clear zone is preserved, flexibility of the anterior leaflet is maintained and the dehiscence of suture line can be avoided. By enhanced stay suture (ESS), though the posterior leaflet and posterior annuals are drawn to the anterior leaflet, the posterior leaflet is compressed and the posterior leaflet height is also slightly reduced. RZT can also reduce excessed degenerated rough-zone of anterior leaflet by resection. These factors may contribute to prevention of occurrence of SAM. The outcomes of this procedure for 25 and 24 diseased anterior mitral and diseased bileaflets, respectively, between February 2007 and January 2013 were favorable. There is no case of suture dehiscence and SAM.

In RZT, resected area of the anterior leaflet was small. We have data for sizes of resected obtuse triangle segments (n = 35). Mean height of the triangles was 4.7 ± 1.9 mm and mean base length of the triangles was 12.8 ± 6.7 mm. However, the resection and suture components of the technique confer risk of creating functional mitral stenosis. It was the only concern about RZT and we had to research about it.

### Evaluation of severity of mitral valve stenosis

Stress (exercise or drug) echocardiography is useful for evaluating mitral valve stenosis [4] and the systolic pulmonary artery pressure at stress is an important parameter for preserving tolerance to exercise [11]. However, few reports have described stress (drug or exercise) echocardiography for evaluating mitral valve function after repair [12,13]. In this study, we applied dobutamine stress echocardiography to evaluate mitral valve function after repair using RZT. The echocardiographic findings of cardiac stress imposed by treadmill or ergometer exercise [14] or by administering drugs such as dobutamine are similar [15]. Since cardiovascular disease occurs in relatively elderly patients, we considered that exercise might not impose sufficient stress because of conditions such as gonarthrosis and arteriosclerosis obliterans. We therefore imposed stress during the echocardiographic study using dobutamine. Several investigators have adopted a maximum dobutamine dose of 40 μg/Kg/min in the DSE protocol [16,17], but dangerous arrhythmias caused some patients to abandon the study [17-19]. Some patients reached the target heart rate at that dose and 10 μg/Kg/min was sufficient to generate cardiotonic action [20]. In fact, one study has limited the maximum dobutamine dose to 20 μg/Kg/min in DSE for evaluating mitral valve stenosis [21]. Safety was our top priority and thus we selected a maximum dobutamine dose of 20 μg/Kg/min.

### Considerations for the results from this study

**Figure 7:**
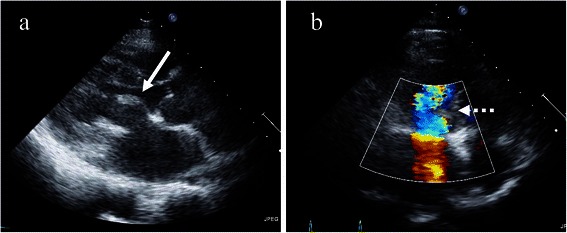
**Postoperative echocardiographic images of anterior mitral leaflet (AML) plasty. (a)** Long axis view. Sclerotic tissue remains in AML (white arrow). **(b)** Four chamber view. Mosaic pattern of transmitral blood flow near sclerotic tissue of AML on color Doppler image suggests that direction of transmitral blood flow became distorted by surging against sclerotic AML tissue (dotted arrow).

The MVA data were similar between the AML and PML groups, and slightly lower in the bileaflet group, but MVA increased at peak stress in all groups. Some investigators have found that MVA was overestimated at tachycardia in the PHT method [22,23], whereas Agricola et al. found that MVA was increased at exercise stress [12], indicating that a reserve mitral valve area might remain to increase cardiac output. The ACC/AHA criterion for severe mitral stenosis is MVmeanPG >15 mmHg or sPAP > 60 mmHg on stress echocardiography and they recommend considering surgery [4]. No pathological findings to fulfill the criterion mentioned above were found in our patients after mitral valve repair or in healthy controls. However, MVmeanPG was somewhat elevated (>10 mmHg) in two patients in the AML group (a1, a7) and one each in the PML (p2) and bileaflet (b2) groups. The MVA of one patient (a1) in the AML and of one (b2) in the bileaflet group were relatively small (1.8 and 2.3 cm^2^, respectively), which might have affected the MVmeanPG. However, the MVA was 3.0 cm^2^ in the other patient (a7) in the AML group and in one patient (p2) in the PML group, and their MVmeanPG was relatively high. The echocardiographic findings of patient a7 in the AML group indicated sclerotic changes at the anterior leaflet and curved transmitral blood flow due to surging against sclerotic AML tissue (Figure 7). Patient p2 in the PML group had sclerotic changes at the posterior leaflet and transmitral blood flow was directed towards the anterior leaflet. Color Doppler images showed a mosaic pattern near the AML suggesting that persistent sclerotic change or the direction of the transmitral flow affected MVmeanPG regardless of the MVA.

The MVmeanPG at rest and peak stress was significantly lower in healthy control than all groups after mitral valve repair. In mitral valve repair groups, the MVA was significantly reduced compared with healthy controls. Since MVA tends to decrease after mitral valve repair [24], not only control of regurgitation, but also functional preservation of the mitral valve should be carefully considered in the future.

We compared our results with those of other echocardiographic findings after mitral valve repair. Zucca et al. repaired mitral valve anterior leaflet prolapse by implanting artificial chordae. Their value for the mean MVA was 2.7 cm^2^ (2.2 – 3.0) [25]. Giovanni et al. performed DSE for patients with Barlow’s disease who had been treated by mitral valve repair (AML: implanting of artificial chordae, PML: quadrangular resection for PML) [11]. Their values for mean MVA, MVmeanPG (rest/stress) and sPAP (rest/stress) were 2.7 ± 0.7 cm^2^, 2.7/6.3 and 33.5/41.6 mmHg, respectively. Agricola et al. repaired mitral valves using the edge-to-edge procedure and investigated outcomes using exercise echocardiography [13]. Their values for mean MVA, MVmeanPG (rest/stress) and sPAP (rest/stress) were 3.2 ± 0.6 cm^2^, 2.8/4.6 and 22.8/28.2 mmHg, respectively. Our findings after RZT for AML prolapse for MVA (rest), MVmeanPG (rest/stress) and sPAP (rest/stress) were 2.8 ± 0.4 cm^2^, 3.3 ± 1.1/7.4 ± 4.1,and 25.7 ± 4.7/49.1 ± 4.1 mmHg, respectively. Although our sPAP values were relatively high, they were similar to those of healthy controls. Thus, we considered that our values were equivalent to those reported, and that the RZT procedure is an appropriate and feasible technique.

### Limitations

All prescribed drugs were continued during the study to consider the safety of the patients. The study cohort was small, because many patients had a history of serious diseases. Because of the nature of echocardiography, not all relevant parameters were measured and some were impossible to measure.

## Conclusions

The Dobutamine stress echocardiography study for RZT revealed mitral valvular function reserve and no pathological obstruction. Novel mitral valve repair technique; RZT is simple, safe and reliable procedure.

## Abbreviations

AML: Anterior mitral leaflet
CO: Cardiac output
dBP: Diastolic blood pressure
DSE: Dobutamine stress echocardiography
EF: Ejection fraction
ESS: Enhanced stay sutures
LVEDV: Left ventricular end diastolic volume
LVESV: Left ventricular end systolic volume
MVA: Mitral valve area
MR: Mitral valve regurgitation
MVmaxPG: Mitral valve maximum pressure gradient
MVmeanPG: Mitral valve mean pressure gradient
p: P value
PML: Posterior mitral leaflet
QR: Quadrangular resection
RZT: Rough-zone trimming
sBP: Systolic blood pressure
sPAP: Systolic pulmonary artery pressure
